# Analysis of research output parameters: Density equalizing mapping and citation trend analysis

**DOI:** 10.1186/1472-6963-9-16

**Published:** 2009-01-27

**Authors:** Beatrix Groneberg-Kloft, Cristian Scutaru, Axel Fischer, Tobias Welte, Carolin Kreiter, David Quarcoo

**Affiliations:** 1 Otto-Heubner Centre, Charité-Universitätsmedizin Berlin, Free University Berlin and Humboldt-University Berlin, Berlin, Germany; 2 Department of Respiratory Medicine, Hannover Medical School, Hannover, Germany; 3 Institute of Occupational Medicine, Charité-Universitätsmedizin Berlin, Free University Berlin and Humboldt-University Berlin, Berlin, Germany

## Abstract

**Background:**

Burden of disease studies indicate major socio-economic burdens since many years. They should be used for the allocation of funding. However, imbalances are present in funding policies and therefore benchmarking becomes increasingly important in health services research.

**Methods:**

The present study assessed benchmarking approaches. Using large data base analyses, research was analyzed for different health research output parameters. The fields of cardiovascular and respiratory medicine served as models to assess irregular patterns of health research. For visualization, density equalizing mapping procedures were used.

**Results:**

Specific areas of major research activity were identified for European countries and large differences were found. Spatial distribution of published items for cardiac and cardiovascular systems differed in comparison to the distribution for the respiratory system. In general, large countries dominated the overall number of published items. When qualitative measures such as citation analysis were assessed, differing results were achieved. In this category, mostly Scandinavian countries dominated.

**Conclusion:**

The present approach of comparative output benchmarking can be used to assess institutional operating figures at the national and international level and to analyze imbalances in health and research funding.

## Background

Respiratory and cardiovascular diseases play a prominent role in burden of disease studies [1] and the global burden studies indicate major socio-economic burdens of these diseases since many years [2].

Disease burden can be defined as the impact of a health problem in an area measured by financial cost, mortality, morbidity, or other indicators. The burden is often quantified in terms of Disability-adjusted life years (DALYs) [3] or Quality-adjusted life years (QALYs) [4,5]. They combine the burden due to both death and morbidity into one index. This allows the comparison of the disease burden in relation to various risk factors or diseases.

With regard to the different diseases listed in the burden of disease ranking, respiratory diseases play a prominent role [1]. In this respect, four of the top ten listed diseases are disorders of the respiratory tract [2]. In view of the anticipated socio-economic burden, it should be expected that there are major health system and research investments into this field of medicine in developed countries. Next to respiratory diseases, disorders of the cardiovascular system also play a prominent role in health systems of industrialized countries and major funding is directed to the treatment and research in this area.

In contrast to these clear socio-economic features, research funding policy is partly controversy in many countries. A variety of publications addressed these issues for areas of medicine and research which are heavily funded by governmental and non-governmental sources. These areas are i.e. cardiovascular medicine [6], neurosciences [7], gastroenterology [8], genetics [9], stem cell research [10-12], or rehabilitation sciences [13,14].

Reviewing the diversity of policies in Europe [15] and general statements [16-20], it is obvious that studies are urgently needed that compare input and output in socio-economic important fields of medicine in order to evaluate funding policy. To meet this goal systematic scientometric analysis of the existing research data in one field has been undertaken in the recent years [21,22]. To handle the enormous amount of data a method has been developed to illustrate the findings in an appealing way. Density-equalizing maps alter the size of regions according to a parameter found in this region, like population or incidence of a disease. Thereby maps are created with easy accessible visual information about the given parameter in a geographic context [23].

The present study was conducted to evaluate different research output parameters. The two important fields of respiratory and cardiovascular diseases which are ranked high in the burden of diseases studies were chosen as target fields. Europe was used as model since there are various centers related to cardiology and respiratory medicine.

## Methods and design

### Data source

Data was retrieved from the biomedical database Web of Science provided by the Thomson Institute for Scientific Information (ISI) [24].

### Search strategies

For the different searches, phrases joined together with Boolean operators, i.e. AND, OR and NOT were used.

### Time frame

A time frame was set and all entries between the years 1900 and 2007 were analyzed. The year 2008 was not included since entries for 2008 are still not terminated in 2008. Some data were added up to five year periods (1973–1977; 1978–1982; 1983–1987; 1988–1992; 1993–1997; 1998–2002; 2003–2007) to provide an overview over a larger period of time.

#### Number and origin of publications

The phrases heart* OR cardiac* OR cardio* combined with the names of the European Union countries and Switzerland were used to find articles related to the cardiac system. Using the filter function from ISI Web only articles which were assigned to the subject category "*Cardiac & Cardiovascular system*" were filtered and analyzed. Analogue, using the search words lung* OR Airway* OR Respirat* or pneumo* and respectively the subject area "*Respiratory system*", provided the respiratory articles. All publication that were retrieved using the below mentioned search strategies that were published in the investigated timeframe were used.

### Quality analysis: Number of citations in relation to origin of publications

To provide data on the quality of the published items, the citation information of the articles was used for the articles that were identified in the previous step. This method was used to assess the average citations per item, indicating the average number of citing articles for all items in the set. It is the sum of the times cited divided by the number of results found. In specific, every single of the identified 89604 articles in the category "cardiac and cardiovascular systems" and the 36322 in the category "respiratory systems" were analyzed for the number of its citations. Values were displayed with the introduction of a threshold of at least 30 published items per country in order to filter out mavericks.

### Density-equalizing mapping

Density-equalizing mapping was used according to a recently published method [23]. The specific calculations are based on Gastner and Newman's algorithm. In brief, territories were re-sized according to a particular variable, i.e. the number of published items or the citations. For the re-sizing procedure the area of each country was scaled in proportion to its total number of published items or citations [23].

### Statistical analysis

Correlation between publication number or citation rate and socioeconomic variables were calculated by using the Pearson correlation analysis in SPSS 17.0. All parameters were first checked for normal distribution using the non-parametric Kolmogorov-Smirnov-test. The gross domestic product was correlated to the purchasing power parity (IMF ). The data for population was retrieved from the WHO (WHOSIS ).

## Results

### Published items in cardiac medicine

**Figure 1:**
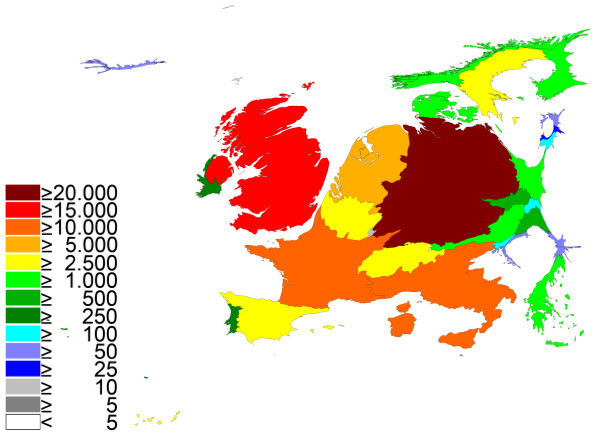
**Spatial distribution of published items using density-equalizing mapping: cardiac and cardiovascular systems**. Color coding encodes total number of published items per country.

Large differences between the various European Union countries were found (Table 1). In specific, Germany published 23% of all items in "cardiac and cardiovascular systems". Thus the relative proportion of Germany increased in the density equalizing score (Fig. 1). Germany was followed by the UK (19.67%), France (12.06%), Italy (11.76%), the Netherlands (7.85%) and Spain (4.31%). All other countries contributed with 25.14%. Due to international cooperation, multiple assignment of an article occured.

**Table 1:** Country-specific total number of published items in the category "cardiac and cardiovascular systems" (89604 identified articles in 131 journals analyzed)

	**Country**	**Published items cardiac system**
1	Germany	20612
2	Great Britain	17631
3	France	10813
4	Italy	10543
5	Netherlands	7037
6	Spain	3870
7	Sweden	3516
8	Belgium	3198
9	Switzerland	3110
10	Denmark	1758
11	Austria	1741
12	Finland	1721
13	Greece	1671
14	Norway	1655
15	Poland	1199
16	Hungary	747
17	Czech Republic	614
18	Ireland	353
19	Portugal	317
20	Slovakia	218
21	Lithuania	144
22	Slovenia	125
23	Romania	88
24	Croatia	87
25	Estonia	80
26	Iceland	69
27	Bulgaria	67
28	Latvia	36
29	Luxembourg	13
30	Andorra	0
31	Liechtenstein	0

### Published items in respiratory medicine

**Figure 2:**
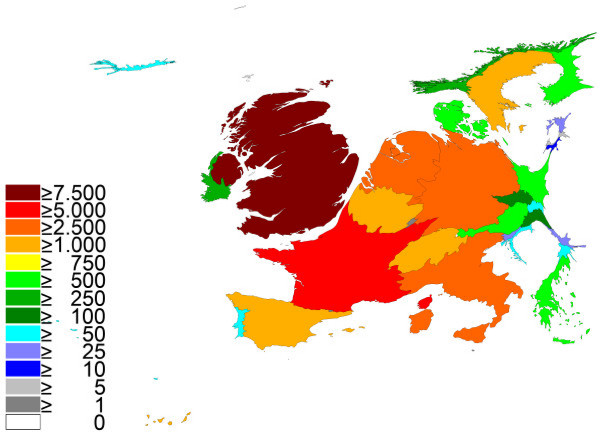
**Spatial distribution of published items using density-equalizing mapping: respiratory medicine**. Color coding encodes total number of published items per country.

As seen with cardiology, large differences between the European countries were present (Table 2). In specific, UK contributed 27.28% of all published items in the category "respiratory medicine". Thus the relative proportion of the UK increased in the density equalizing score (Fig. 2). The UK was followed by France (16.66%), Germany (12.62%), Italy (10.12%), the Netherlands (7.26%) and Spain (6.37%). All other countries contributed with 26.88%. As previously stated, due to international cooperation, multiple assignment of an article occured.

**Table 2:** Country-specific total number of published items in the category "respiratory system" (36322 identified articles in 58 journals analyzed)

	**Country**	**Published items respiratory system**
1	Great Britain	9908
2	France	6052
3	Germany	4585
4	Italy	3675
5	Netherlands	2636
6	Spain	2313
7	Belgium	1831
8	Sweden	1824
9	Switzerland	1382
10	Denmark	737
11	Finland	695
12	Greece	601
13	Austria	601
14	Poland	513
15	Norway	402
16	Ireland	283
17	Czech Republic	205
18	Hungary	180
19	Portugal	95
20	Slovakia	79
21	Bulgaria	59
22	Iceland	57
23	Croatia	51
24	Romania	47
25	Estonia	46
26	Slovenia	44
27	Lithuania	15
28	Latvia	6
29	Andorra	5
30	Luxembourg	4
31	Liechtenstein	0

### Published items: time course

To evaluate the kinetic of publication we analyzed the total publication output of the countries in five years intervals (see additional files 1 and 2). For both fields a continuing rise of publication was found with a tendency to increased progression after the year 1997. Except for one case (respiratory systems, Poland 1983–1987) we did not detect great country based variations of output. The increase of publications was also visualized for respiratory medicine in one-year steps using density-equalizing calculations (see additional files 3).

### Citations per published items in cardiac medicine

**Figure 3:**
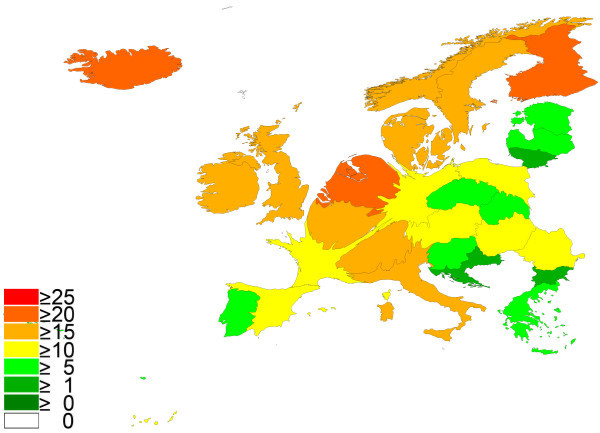
**Spatial distribution of citations per published item: cardiac and cardiovascular systems**. Density equalizing mapping with threshold of 30 published item per country. Color coding encodes average citation per published item per country.

To provide qualitative data the citation report analysis was used for all articles that were found in the previously assigned published items. With a threshold of 30 published items (Table 3), three Scandinavian countries Iceland and the Netherlands were ranked in the first 5 positions (Finland with a mean citation per item of 23.57 followed by Iceland (20.45), the Netherlands (20.15), Sweden (19.80) and Denmark (19.29). Germany that led the overall number of published items analysis was ranked 12 with a value of 13.36. When the data of the citation analysis was transferred to density-equalizing mapping and the threshold of at least 30 publications was used, the resulting map is presented in Fig. 3.

**Table 3:** Country-specific mean citation per published item in the category "cardiac and cardiovascular systems"

**Cardiac system**		
	**Country**	**Mean citation per Item (threshold 30 published items)**
1	Finland	23.57
2	Iceland	20.45
3	Netherlands	20.15
4	Sweden	19.80
5	Denmark	19.29
6	United Kingdom	17.47
7	Ireland	17.33
8	Belgium	17.29
9	Norway	17.17
10	Switzerland	16.50
11	Italy	15.71
12	Germany	13.36
13	Austria	12.73
14	France	12.47
15	Hungary	10.71
16	Spain	10.69
17	Romania	10.40
18	Poland	10.11
19	Czech Republic	9.51
20	Portugal	8.52
21	Greece	8.26
22	Estonia	8.05
23	Slovenia	7.72
24	Latvia	7.39
25	Slovakia	6.59
26	Croatia	4.69
27	Lithuania	3.40
28	Bulgaria	2.60
29	Andorra	0.00
30	Liechtenstein	0.00
31	Luxembourg	0.00

### Citations per published items in respiratory medicine

**Figure 4:**
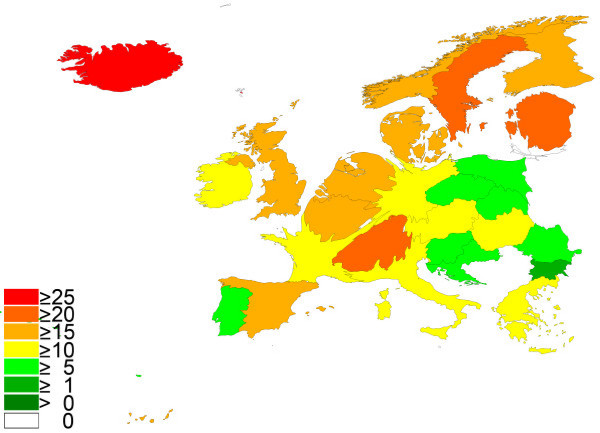
**Spatial distribution of citations per published item: respiratory system**. Density equalizing mapping with threshold of 30 published item per country. Color coding encodes average citation per published item per country.

The citation analysis for the articles published in the respiratory system category led to slightly differing results (Table 4). Here, Iceland dominated the ranking with a relation of 27.70 citations per item followed by Sweden (21.16). Switzerland were ranked 3 (20.90), Estonia 4 (20.59), and Finland 5 (19.74. United Kingdom placed 7^th ^with a value of 19.03 before Germany which placed 15^th ^(13.57). Density-equalizing mapping produced a continental shape presented in Fig. 4.

**Table 4:** Country-specific mean citation per published item in the category "respiratory system"

**Respiratory system**		
	**Country**	**Mean citation per item (threshold 30 published items)**
1	Iceland	27.70
2	Sweden	21.16
3	Switzerland	20.90
4	Estonia	20.59
5	Finland	19.74
6	Netherlands	19.22
7	United Kingdom	19.03
8	Denmark	17.75
9	Belgium	17.23
10	Norway	16.39
11	Spain	15.64
12	Italy	14.91
13	Ireland	14.48
14	France	13.71
15	Germany	13.57
16	Greece	12.99
17	Austria	11.74
18	Hungary	11.08
19	Czech Republic	9.54
20	Poland	9.21
21	Portugal	9.07
22	Romania	8.72
23	Slovakia	7.65
24	Slovenia	7.64
25	Croatia	6.71
26	Bulgaria	2.90

### Cooperation analysis

**Figure 5:**
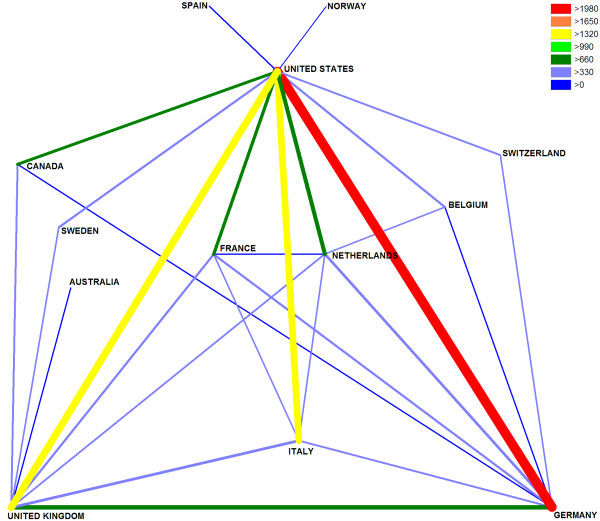
**Cardiac system international cooperations**. Only cooperation values over 250 articles are shown for readability reasons. Color coding encodes amount of international cooperation.

**Figure 6:**
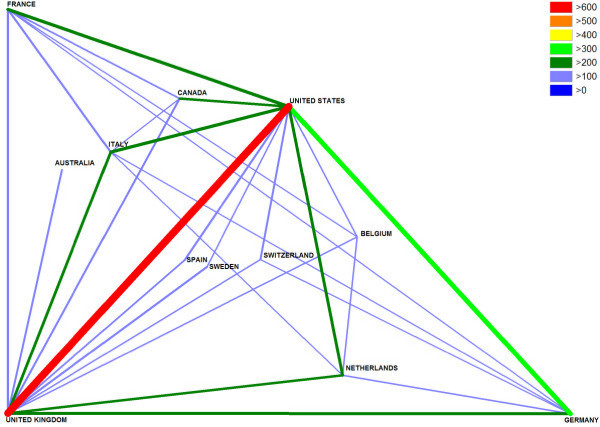
**Respiratory system international cooperations**. Only cooperation values over 100 articles are shown for readability reasons. Color coding encodes amount of international cooperation.

Country identity analysis demonstrated that the USA and Germany are the leading countries concerning bilateral cooperation in cardiac medicine with a total of 1990 cooperation articles (Fig. 5). For respiratory medicine USA and UK lead the international cooperation with 616 articles (Fig. 6). A country specific analysis of cooperation for Germany and UK is presented in the additional files 4 and 5.

### Correlation of bibliometric with socioeconomic data

For cardiac medicine a strong correlation was found for the number of articles and the gross domestic product (r = .952, p = 0.01) and population (r = .889, p = 0.01). The average citation per item neither correlate with population nor with the gross domestic product.

For respiratory medicine a strong correlation was found for the number of articles and the gross domestic product (r = .953, p = 0.01) and population (r = .926, p = 0.01). The average citation per item neither correlate with population nor with the gross domestic product.

## Discussion and conclusion

The current policy settings for health system and research funding allocation urgently needs to be reviewed, since there may be imbalances present regarding socio-economic features. Research evaluation and policy projects in countries such as the US or Australia have demonstrated the existence and nature of this problem in their countries but there is still a lack of a scientific approach for many areas of research especially in European countries where only little data is available [25-27]. Therefore, the present study aimed to assess different output parameters using the fields of respiratory and cardiovascular medicine. In contrast to some of the recent studies with similar a goal we choose a category-, rather than journal-based searching strategy thereby avoiding the omittance of journals that have changed their status [28,29] (e.g. being renamed or closed down). Also, this approach bears the advantage of including important articles that have been published in journals that are of more general orientation, never the less containing important new diagnostic, pathophysiological or therapeutical aspects of respiratory, respectively cardiac diseases. For the general output parameter of published items major differences were found between single countries: The UK led the field of respiratory medicine with 9908 published items in total. In this category, Germany was ranked third behind France (6052) with less than half the amount of published articles (4585). By contrast, in the category cardiac and cardiovascular systems, Germany ranked #1 with 20612 published items followed by the UK with 17631 and France with 10813 published items. The reasons for these differences may be at least two-fold: 1) It is known that Germany, although economically strong, has a low ratio of respiratory physicians in comparison to other European countries [30]. Therefore, a lower number of specialists may lead to a lower number of studies. 2) The number of full professorships and department chairs for respiratory medicine (C4/W3 salary level) is disproportional in Germany in comparison to other countries: There are 37 medical school department chairs for cardiology but only 7 for respiratory medicine. This imbalance might also be a reason for the lower number of entries in the database. Reasons for the low numbers of respiratory medicine chairs at German medical school may base on traditional and economic interests: 1) Historically, respiratory disorders in the times of tuberculosis were treated in remote hospitals but not in university medical school departments [31,32]. Therefore, respiratory medicine might have been underrepresented at the faculties. 2) Economically, diagnostic procedures in cardiology such as left heart catheters bring a larger financial benefit than respiratory diagnostic procedures [33]. Therefore, medical faculties may be biased to the direction of cardiologic professorships due to economical reasons. Future studies should analyze these imbalances in closer detail.

As a second approach in the present study, the average citation for each published item was calculated for each country in both categories. Since a low number of published items with no first or senior authorship could lead to a major distortion of the real scientific output, a threshold was included to the density equalizing calculations leading to a more proportional visualization with regard to the overall numbers of published items.

The following problems can occur within the present study setting: Database biases. There is a bias towards English-speaking countries. In this respect, the issue of linguistic differences and its effects on publication quantity should be taken into account. In the present data base searches all languages were included. The majority of publications are published in English and it is more difficult for non-English journals to get included in the data bases. Therefore, a higher proportion of scientific publications in languages other than English are not accessible in comparison to English-written publications. Therefore, English speaking countries such as the UK or Ireland should have an advantage. However, it is generally accepted that scientists from non-English speaking countries in Europe publish their high quality research in scientific journals that use English as language. It can therefore be estimated that the number of highly cited publications is relatively low in journals not listed in the presently analyzed two categories of "cardiac and cardiovascular systems" and "respiratory system".

In summary, the present study encompasses a novel approach to assess and compare research output parameters. The study was conducted to provide and interpret quantitative benchmarking data in the European Union. Benchmarking systems basing on research output can be efficiently used to identify individual and regional differences. However, these approaches should be backed up by detailed analyses of socio-economic features to improve European policy settings for research evaluation.

## Competing interests

The authors declare that they have no competing interests.

## Authors' contributions

BGK conceived the scientific study, participated in the design and co-ordination of the study, performed the analysis, and drafted and prepared the manuscript. CS, CK and DQ conceived the scientific study and participated in the interpretation, AF and TW helped to interpret the data. All authors read and approved the final manuscript.

## Pre-publication history

The pre-publication history for this paper can be accessed here:



## Supplementary Material

Additional file 1 **Cardiac system time evolution of published items per country.** The data provided represent the number of published items related to the cardiac system between 1973 and 2007. Click here for file

Additional file 2 **Respiratory system time evolution of published items per country.** The data provided represent the number of published items related to the respiratory system between 1973 and 2007. Click here for file

Additional file 3 **Time-space distribution of published items related to respiratory medicine using density-equalizing mapping and one-year steps**. The film sequence visualizes the total increase in respiratory medicine publications. The color coding encodes the total number of published items per country over the time. Click here for file

Additional file 4 **Radar chart – unilateral cooperation of Germany for cardiac and respectively respiratory systems. **These 2 radar charts represent the unilateral cooperations of Germany for publications related to the cardiac and the respiratory system. Click here for file

Additional file 5 **Radar chart – unilateral cooperation of UK for cardiac and respectively respiratory systems.** These 2 radar charts represent the unilateral cooperations of the UK for publications related to the cardiac and the respiratory system. Click here for file
