# Supplementary figures and images for: Analysis of research output parameters: Density equalizing mapping and citation trend analysis
Source: BMC Health Serv Res. 2009 Jan 27;9:16. doi: 10.1186/1472-6963-9-16 (PMC2672943; doi:10.1186/1472-6963-9-16)

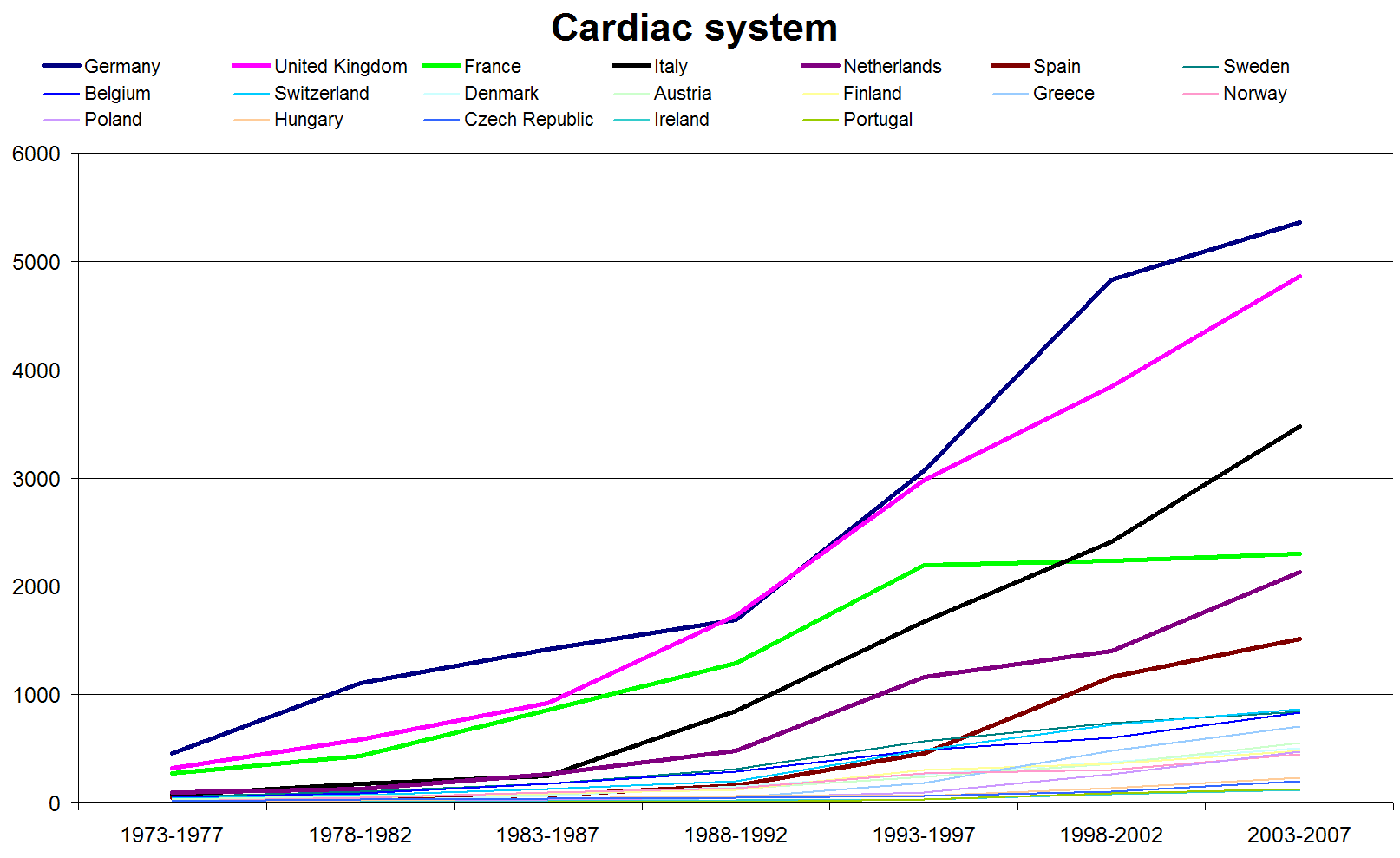

Supplement: Additional file 1 — Cardiac system time evolution of published items per country. The data provided represent the number of published items related to the cardiac system between 1973 and 2007. [file 1472-6963-9-16-S1.tiff]

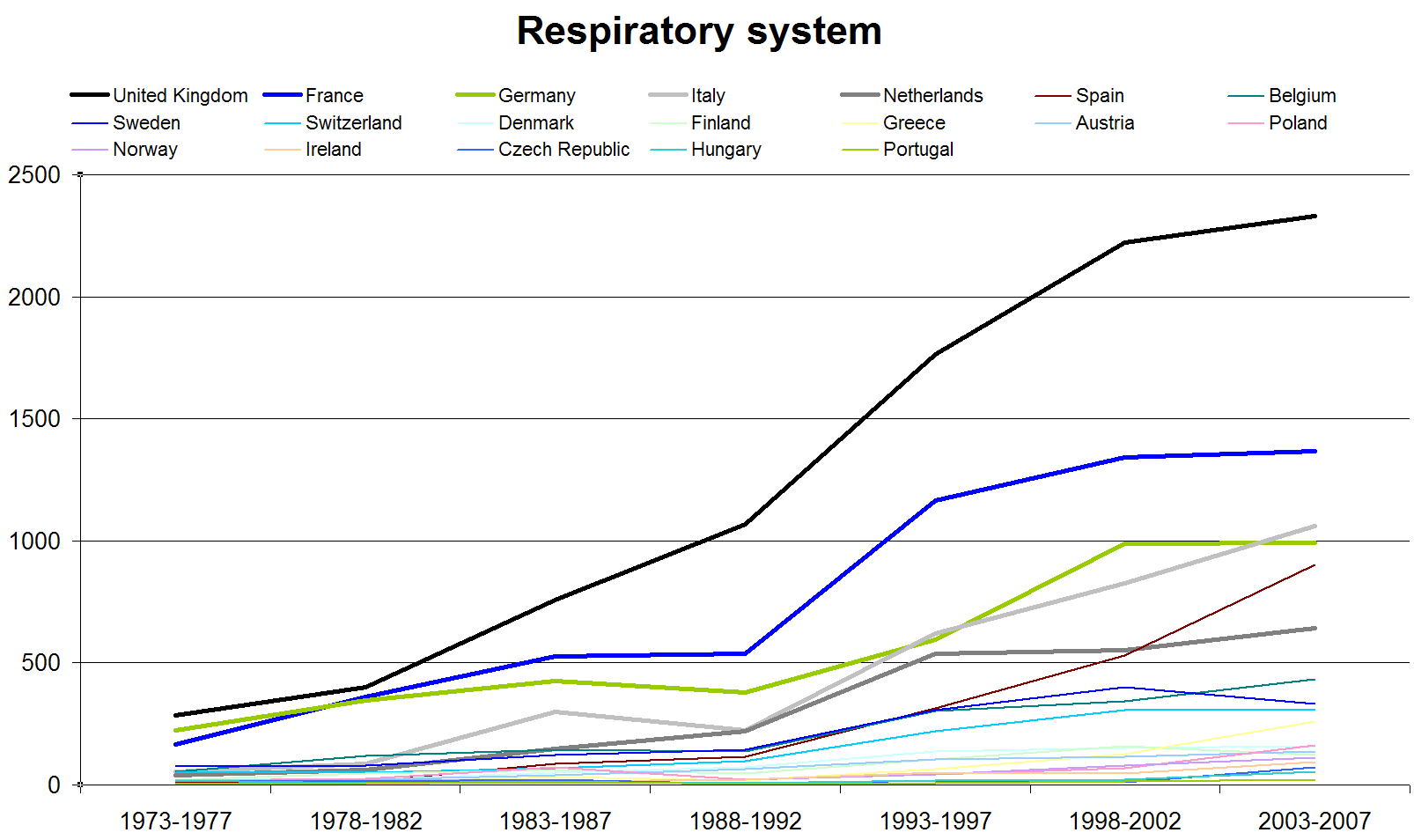

Supplement: Additional file 2 — Respiratory system time evolution of published items per country. The data provided represent the number of published items related to the respiratory system between 1973 and 2007. [file 1472-6963-9-16-S2.tiff]
